# Supplementary material for: MAJA: multivariate Bayesian model for discovery of shared epigenetic pathways across human phenotypes
Source: Bioinform Adv. 2026 Aug 12;6(1):vbag231. doi: 10.1093/bioadv/vbag231 (PMC13587246; doi:10.1093/bioadv/vbag231)
Supplement: Supplementary file 1 [file vbag231_supplementary_data.pdf]

Supplementary information

MAJA: Multivariate Bayesian model for discovery of shared epigenetic pathways across human phenotypes

Ilse Krätschmer, Hannah M. Smith, Daniel L. McCartney, Elena Bernabeu, Mahdi Mahmoudi, Archie Campbell, Janie Corley, Sarah E Harris, Simon R. Cox, Riccardo E. Marioni, Matthew R. Robinson

Table S1: Estimated posterior (co)variances, $\hat{V}$, and 95% confidence intervals (CI) for body mass index (BMI), ratio of high density lipoprotein over total cholesterol (CHL), high blood pressure (BP), depression (DEP), osteoarthritis (OA) and asthma (AT).

**phenotype**	$\hat{\boldsymbol{V}}$	**95% CI**
BMI	0.8182	0.0270
CHL	0.7239	0.0339
BP	0.6877	0.0526
DEP	0.2426	0.0361
OA	0.5750	0.0777
AT	0.2367	0.0345
BMI-CHL	−0.2939	0.0262
BMI-BP	0.1408	0.0274
CHL-BP	−0.0306	0.0303
BMI-DEP	0.0692	0.0232
CHL-DEP	0.0102	0.0321
BP-DEP	0.0328	0.0392
BMI-OA	0.0721	0.0278
CHL-OA	0.0827	0.0397
BP-OA	0.2794	0.0488
DEP-OA	0.0017	0.0260
BMI-AT	0.0392	0.0245
CHL-AT	−0.0096	0.0242
BP-AT	−0.0679	0.0336
DEP-AT	0.0146	0.0106
OA-AT	0.0103	0.0384

Table S2: Estimated posterior (co)variances, $\hat{V}$, and 95% confidence intervals (CI) for years in education (EY), highest qualification in education (EQ), digit symbol (DS), logical memory (LM), verbal fluency (VT) and vocabulary (VO) tests.

**phenotype**	$\hat{V}$	**95% CI**
EY	0.5791	0.0760
EQ	0.7778	0.0750
DS	0.6446	0.0475
LM	0.3232	0.0514
VT	0.4247	0.0647
VO	0.7311	0.1026
EY-EQ	-0.5142	0.0927
EY-DS	0.3121	0.0436
EY-LM	0.2133	0.0480
EY-VT	0.1835	0.0542
EY-VO	0.3284	0.0986
EQ-DS	-0.3799	0.0475
EQ-LM	-0.2549	0.0613
EQ-VT	-0.2371	0.0579
EQ-VO	-0.4254	0.0882
DS-LM	0.2393	0.0368
DS-VT	0.1760	0.0425
DS-VO	0.1109	0.0527
LM-VT	0.0859	0.0483
LM-VO	0.1542	0.0623
VT-VO	0.3082	0.0579

Table S3: Correlations, ρ, calculated from the posterior (co)variances for body mass index (BMI), ratio of high density lipoprotein over total cholesterol (CHL), high blood pressure (BP), depression (DEP), osteoarthritis (OA) and asthma (AT), for the cases where the covariances were different from 0. The upper and lower uncertainties are calculated using the posterior means $\pm$ 95% credible interval.

**phenotypes**	ρ	**lower uncert.**	**upper uncert.**
BMI-CHL	-0.3819	0.0513	0.0474
BMI-BP	0.1877	0.0277	0.0249
BMI-DEP	0.1553	0.0416	0.0352
BMI-OA	0.1051	0.0345	0.0294
BMI-AT	0.0891	0.0522	0.0439
CHL-OA	0.1282	0.0548	0.0458
BP-OA	0.4443	0.0340	0.0278
BP-AT	-0.1683	0.1149	0.0918
DEP-AT	0.0610	0.0415	0.0309

Table S4: Phenotypic or tetrachoric correlations (for dichotomous phenotypes) between body mass index (BMI), ratio of high density lipoprotein over total cholesterol (CHL), high blood pressure (BP), depression (DEP), osteoarthritis (OA) and asthma (AT).

**phenotypes**	**correlation**
BMI-CHL	−0.338
BMI-BP	0.220
BMI-DEP	0.063
BMI-OA	0.105
BMI-AT	0.027
CHL-BP	−0.036
CHL-DEP	−0.036
CHL-OA	−0.017
CHL-AT	0.005
BP-DEP	0.141
BP-OA	0.377
BP-AT	0.008
DEP-OA	0.186
DEP-AT	0.132
OA-AT	0.074

Table S5: Correlations, ρ, calculated from the posterior (co)variances for years in education (EY), highest qualification in education (EQ), digit symbol (DS), logical memory (LM), verbal fluency (VT) and vocabulary (VO) tests, for the cases where the covariances were different from 0. The upper and lower uncertainties are calculated using the posterior means ± 95% credible interval.

**phenotpyes**	$\boldsymbol{\rho}$	**lower uncert.**	**upper uncert.**
EY-EQ	-0.7661	0.2544	0.2022
EY-DS	0.5108	0.0209	0.0174
EY-LM	0.4930	0.0460	0.0344
EY-VT	0.3700	0.0661	0.0497
EY-VO	0.5046	0.0960	0.0731
EQ-DS	-0.5366	0.1232	0.1038
EQ-LM	-0.5083	0.2151	0.1658
EQ-VT	-0.4125	0.1739	0.1351
EQ-VO	-0.5640	0.2086	0.1642
DS-LM	0.5243	0.0215	0.0179
DS-VT	0.3364	0.0485	0.0391
DS-VO	0.1616	0.0666	0.0539
LM-VT	0.2318	0.1116	0.0815
LM-VO	0.3173	0.0949	0.0702
VT-VO	0.5531	0.0269	0.0200

Table S6: Phenotypic correlations between years in education (EY), highest qualification in education (EQ), digit symbol (DS), logical memory (LM), verbal fluency (VT) and vocabulary (VO) tests.

**phenotypes**	**correlation**
EY-EQ	-0.548
EY-DS	0.303
EY-LM	0.220
EY-VT	0.195
EY-VO	0.278
EQ-DS	-0.335
EQ-LM	-0.251
EQ-VT	-0.232
EQ-VO	-0.324
DS-LM	0.308
DS-VT	0.232
DS-VO	0.056
LM-VT	0.153
LM-VO	0.217
VT-VO	0.386

Table S7: Loadings of cognitive tests on intercept. Latent measures of general cognitive function were generated using confirmatory factor analysis in a structural equation modelling (SEM) framework. Levels and change in general cognitive functioning were modelled with latent growth curve model (LGCM) using a Factor of Curves specification. The intercepts were used to indicate a latent intercept of general cognitive function and the test loadings are given. Model fit measures were calculated including confirmatory factor index (CFI = 0.958), Tucker-Lewis index (TLI = 0.957), root mean squared error approximation (RMSEA = 0.029) and the standardised root mean squared residual (SRMR = 0.061).

**Test**	**Intercept term**
Block design	0.702
Matrix reasoning	0.792
Span total	0.642
NART	0.685
WTAR	0.679
Verbal fluency	0.534
Verbal paired associates	0.543
Logical memory	0.615
Digit backwards	0.722
Symbol search	0.788
Digit symbol	0.668
Inspection time	0.491
Four choice reaction time	0.526

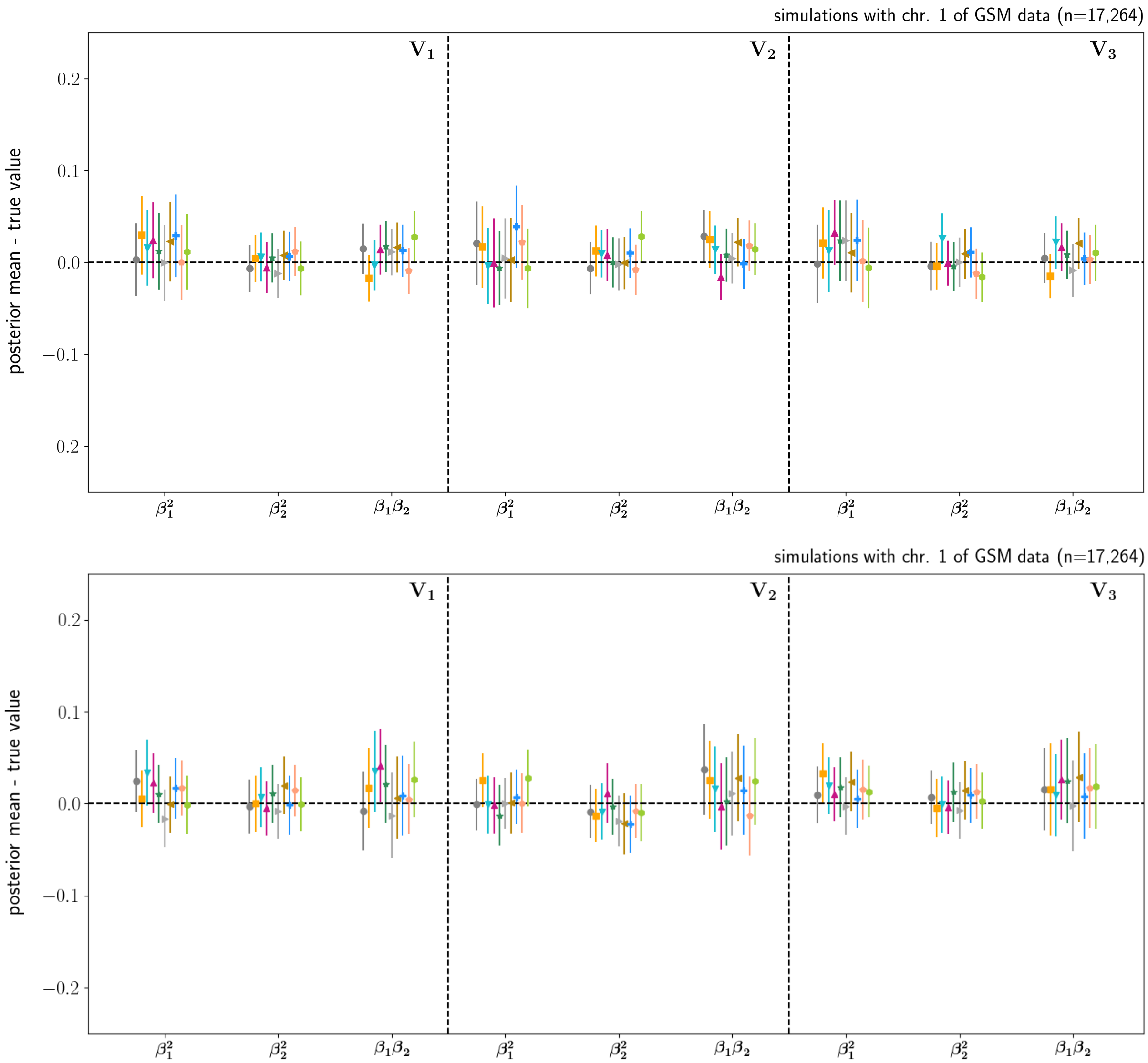

Figure S1: Simulation study results: Posterior mean estimates of the effects (co)variance components subtracted by their true value for each simulated dataset for three different covariance scenarios, denoted as V_1 to V_3, with diag($\mathbf{V}$) = [0.5, 0.2] (top) and [0.3, 0.5] (bottom) and 1000 causal probes. The error bars represent the 95% credible intervals.

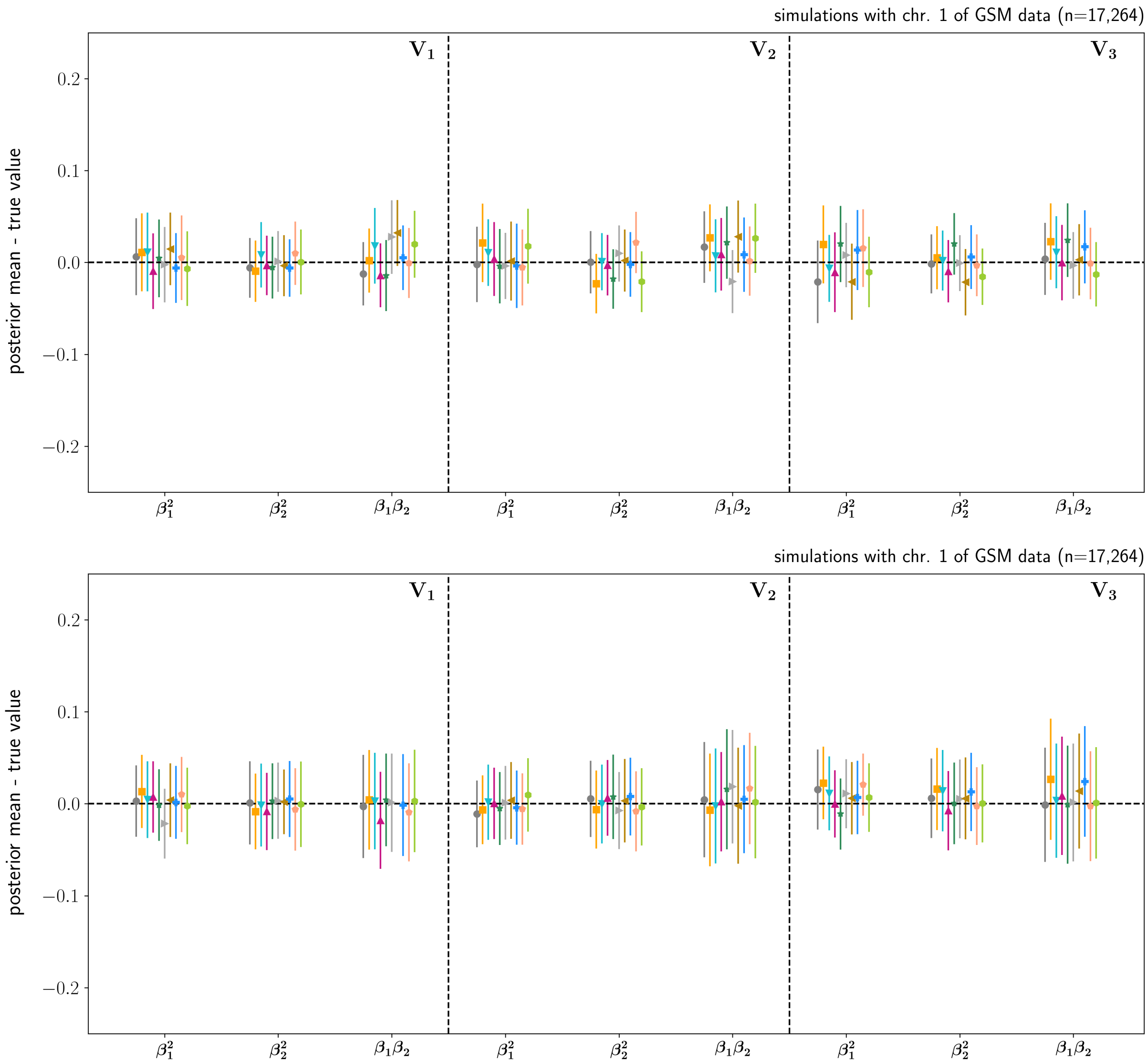

Figure S2: Simulation study results: Posterior mean estimates of the effects (co)variance components subtracted by their true value for each simulated dataset for three different covariance scenarios, denoted as V_1 to V_3, with diag($\mathbf{V}$) = [0.5, 0.4] (top) and [0.5, 0.8] (bottom) and 1000 causal probes. The error bars represent the 95% credible intervals.

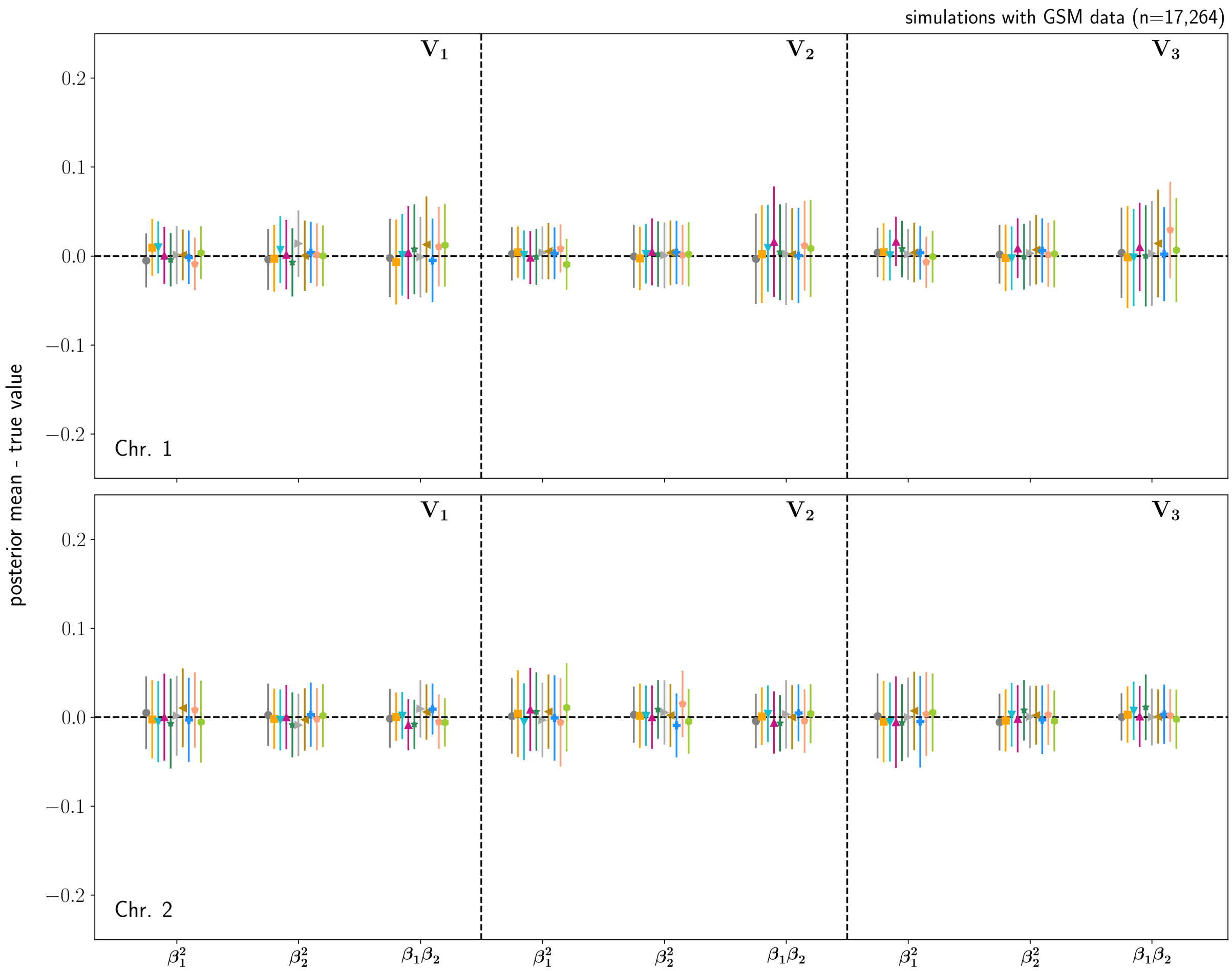

Figure S3: Simulation study results for two groups: Posterior mean estimates of the effects (co)variance components subtracted by their true value for each simulated dataset for three different covariance scenarios, denoted as V_1 to V_3, two groups and 10 simulation replicates (colours). The number of causal probes is set to 1000. The error bars represent the 95% credible intervals.

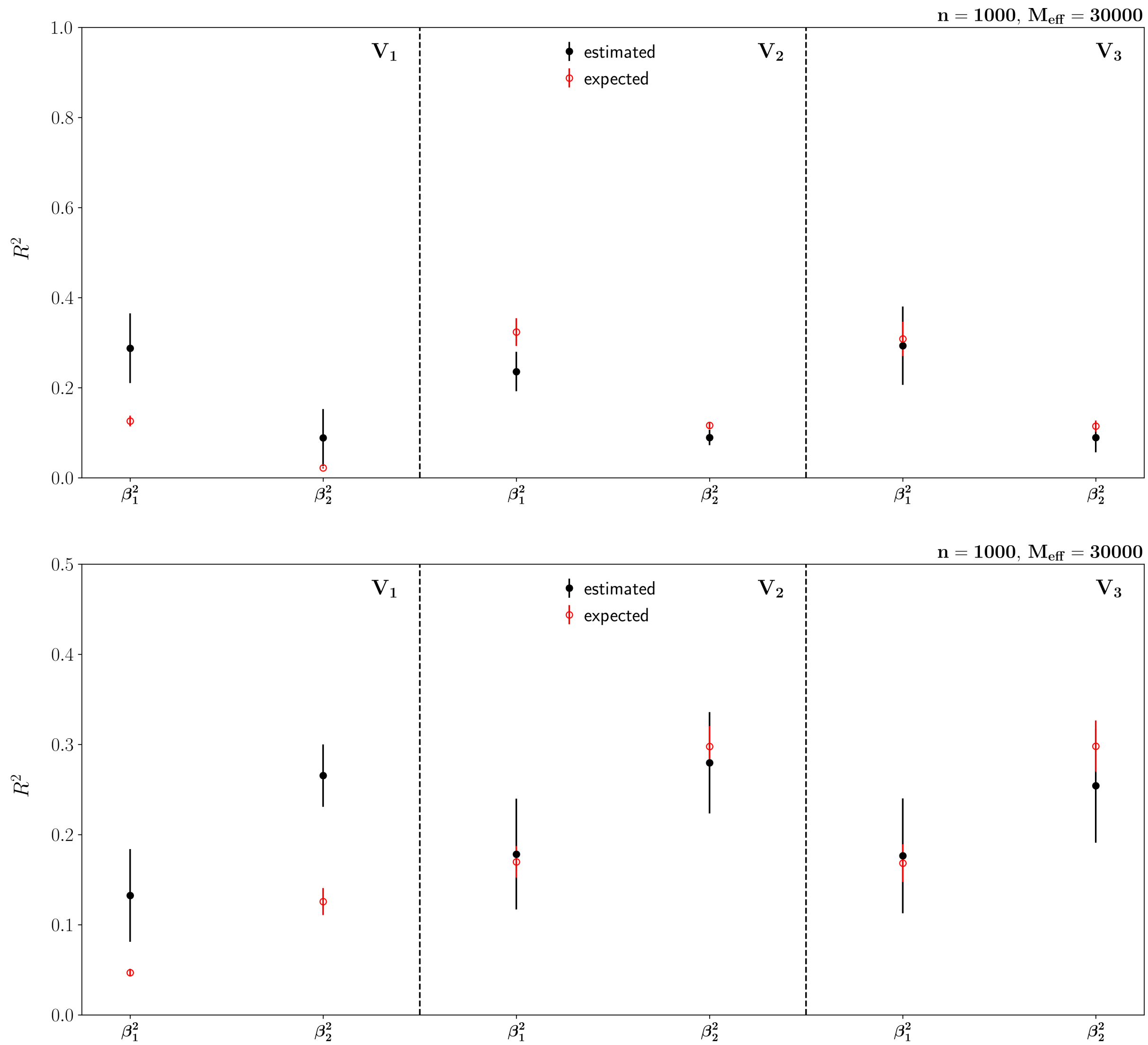

Figure S4: Mean expected and predicted coefficient of determination, R^2, for three covariance scenarios (V_1 to V_3) with diag($\mathbf{V}$) = [0.5, 0.2] (top) and [0.3, 0.5] (bottom), and 10 simulation replicates (colours) with 1000 individuals and 1000 causal probes for each scenario.

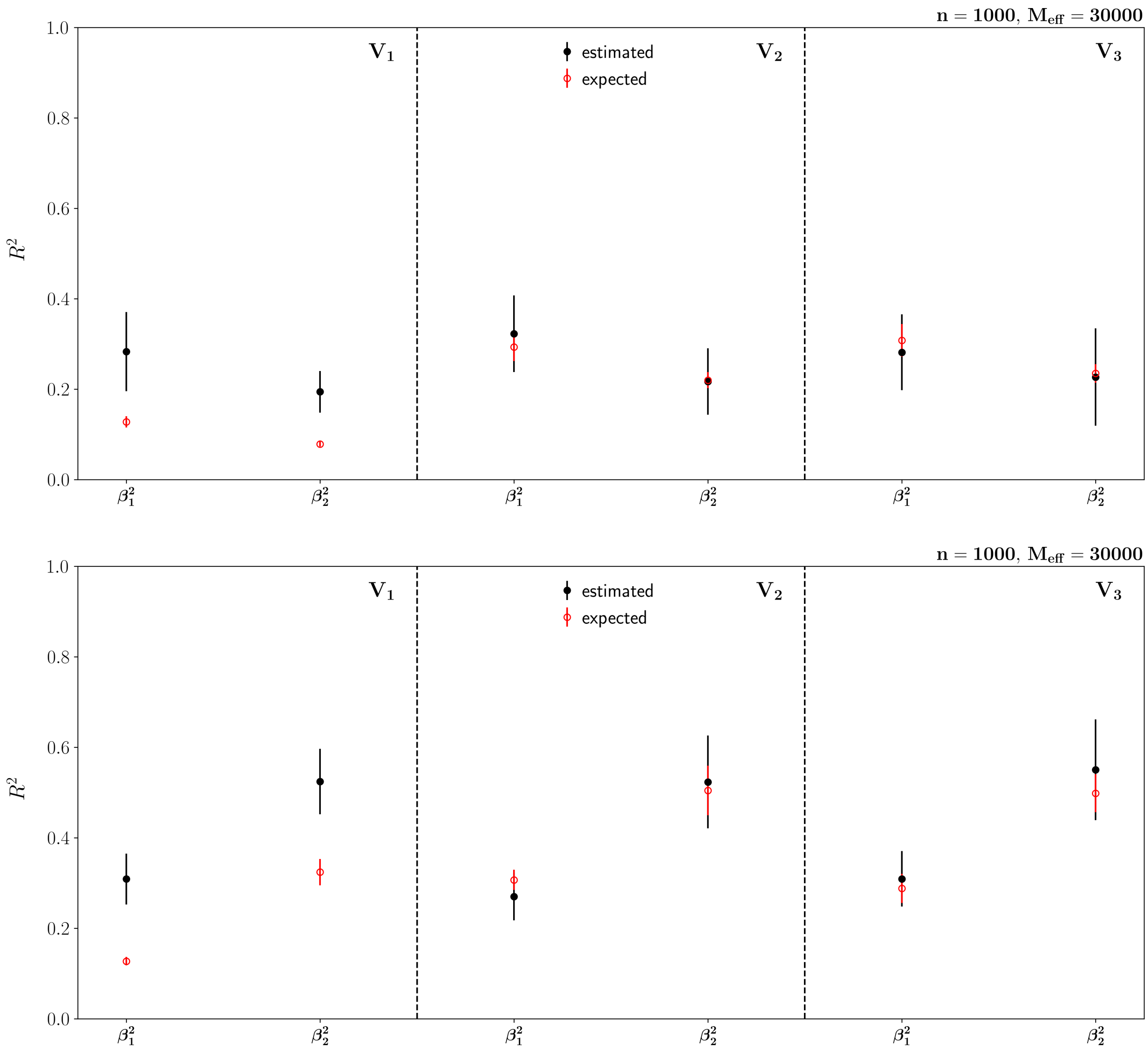

Figure S5: Mean expected and predicted coefficient of determination, R^2, for three covariance scenarios (V_1 to V_3) with diag($\mathbf{V}$) = [0.5, 0.4] (top) and [0.5, 0.8] (bottom), and 10 simulation replicates (colours) with 1000 individuals and 1000 causal probes for each scenario.

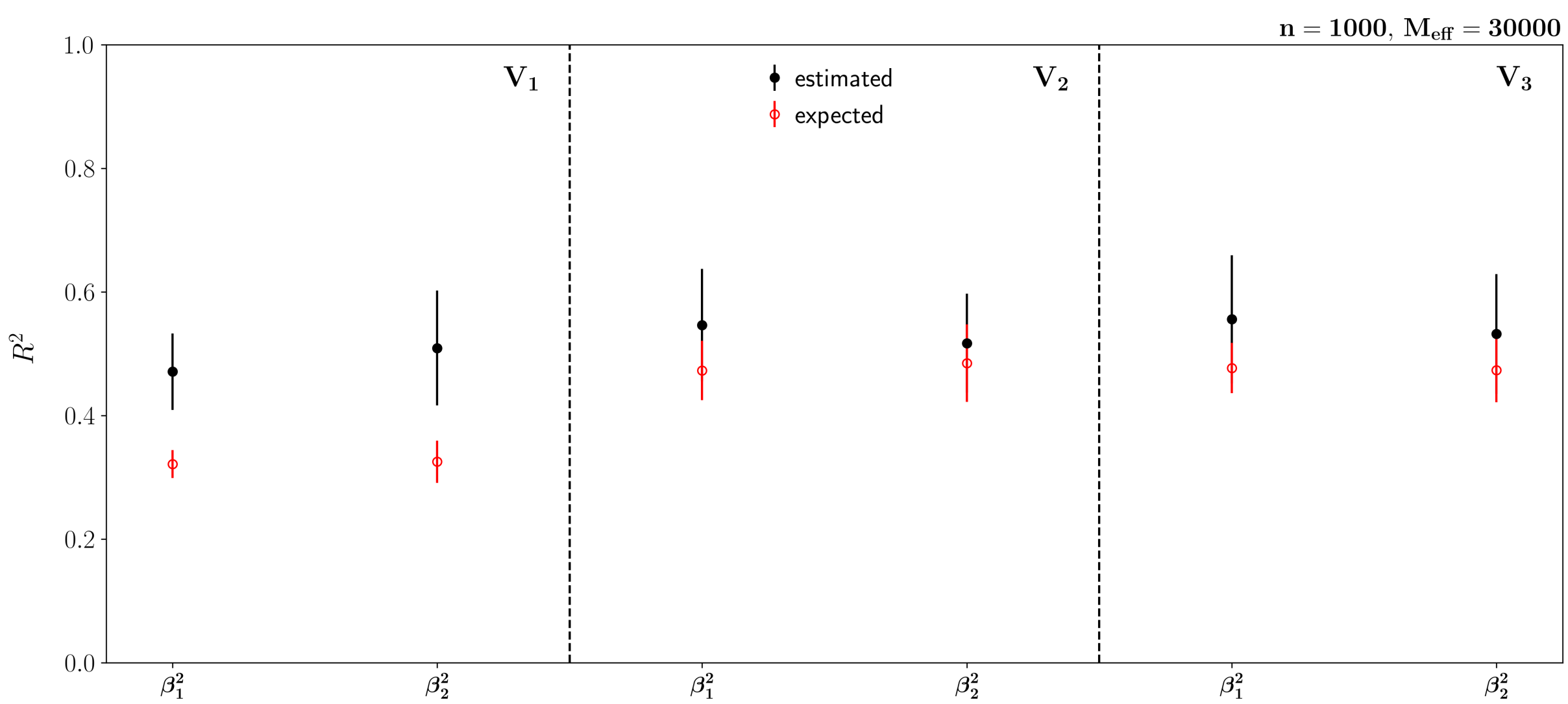

Figure S6: Mean expected and predicted coefficient of determination, R^2, for three covariance scenarios (V_1 to V_3) and 10 simulation replicates (colours) with 1000 individuals and 1000 causal probes, where the effects were estimated for two different groups.

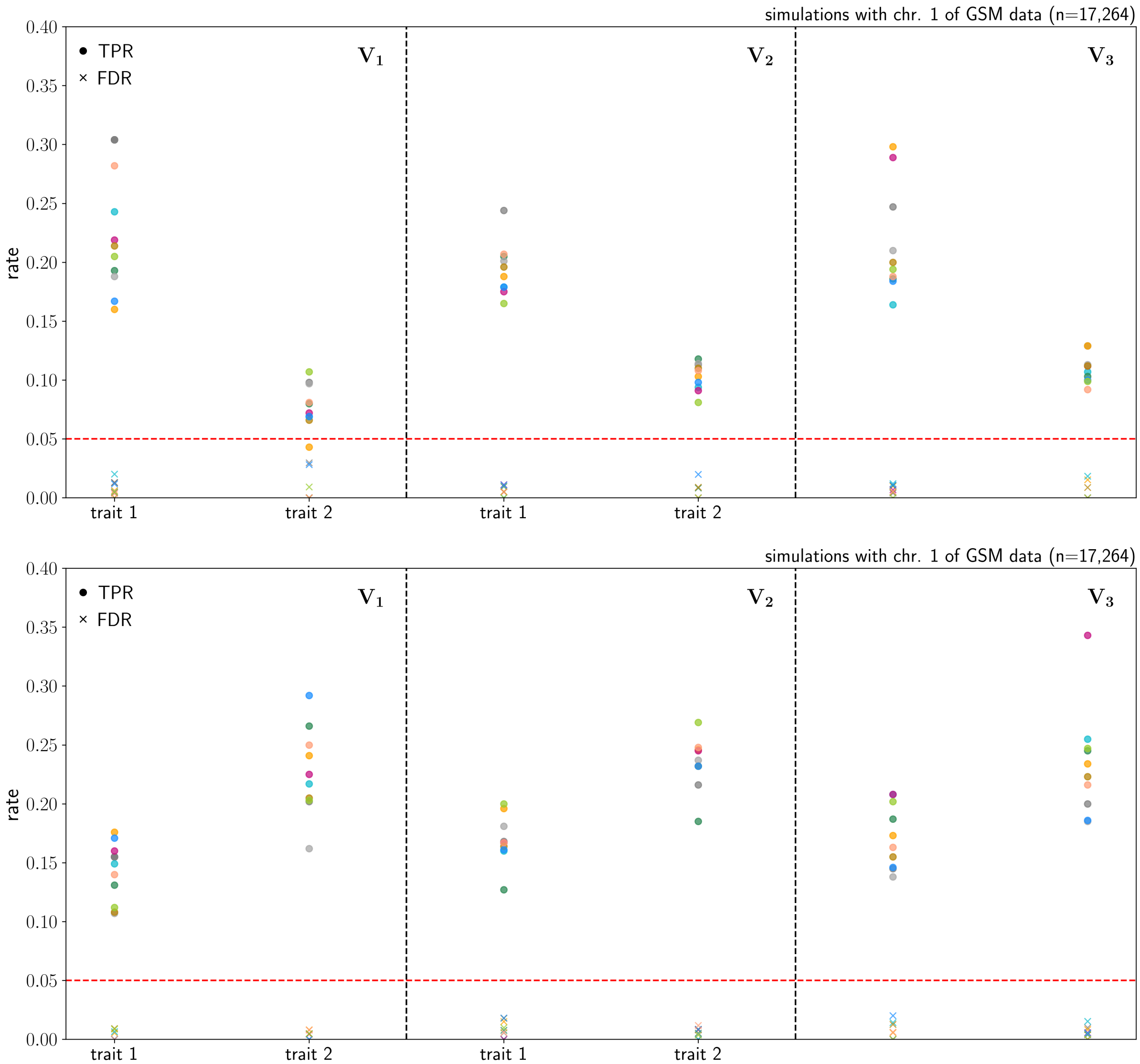

Figure S7: True positive rate (TPR) and false discovery rate (FDR) each for three different covariance scenarios, denoted as V_1 to V_3, with diag($\mathbf{V}$) = [0.5, 0.2] (top) and [0.3, 0.5] (bottom). The red dashed line indicates an FDR threshold of 5%. MAJA controls the FDR well below 2% for all scenarios, with the TPR (power) dependent upon the relationship among the traits.

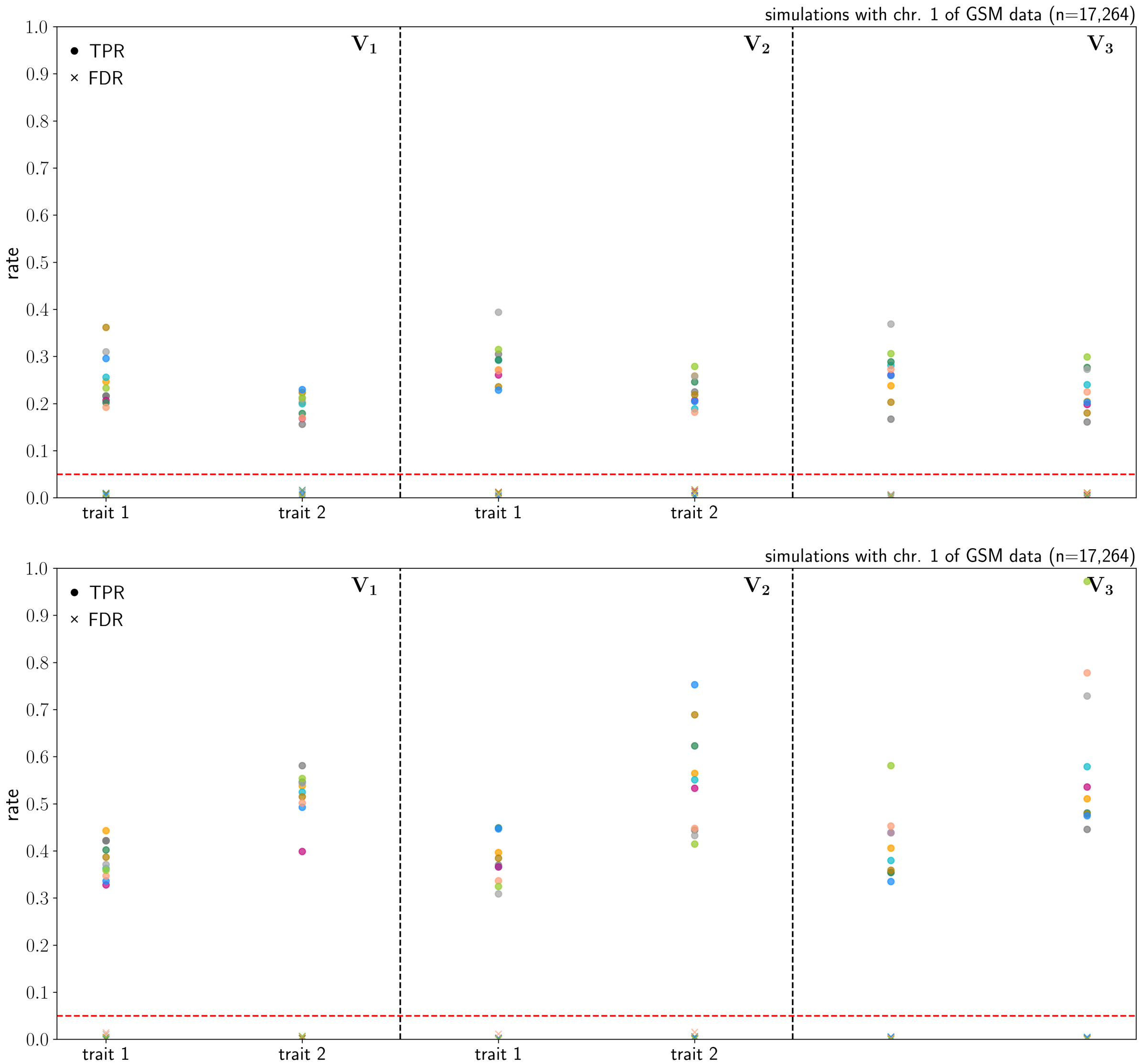

Figure S8: True positive rate (TPR) and false discovery rate (FDR) for two traits estimated for 10 simulations (colours) each for three different covariance scenarios, denoted as V_1 to V_3, with diag($\mathbf{V}$) = [0.5, 0.4] (top) and [0.5, 0.8] (bottom). The red dashed line indicates an FDR threshold of 5%. MAJA controls the FDR well below 2% for all scenarios, with the TPR (power) dependent upon the relationship among the traits.

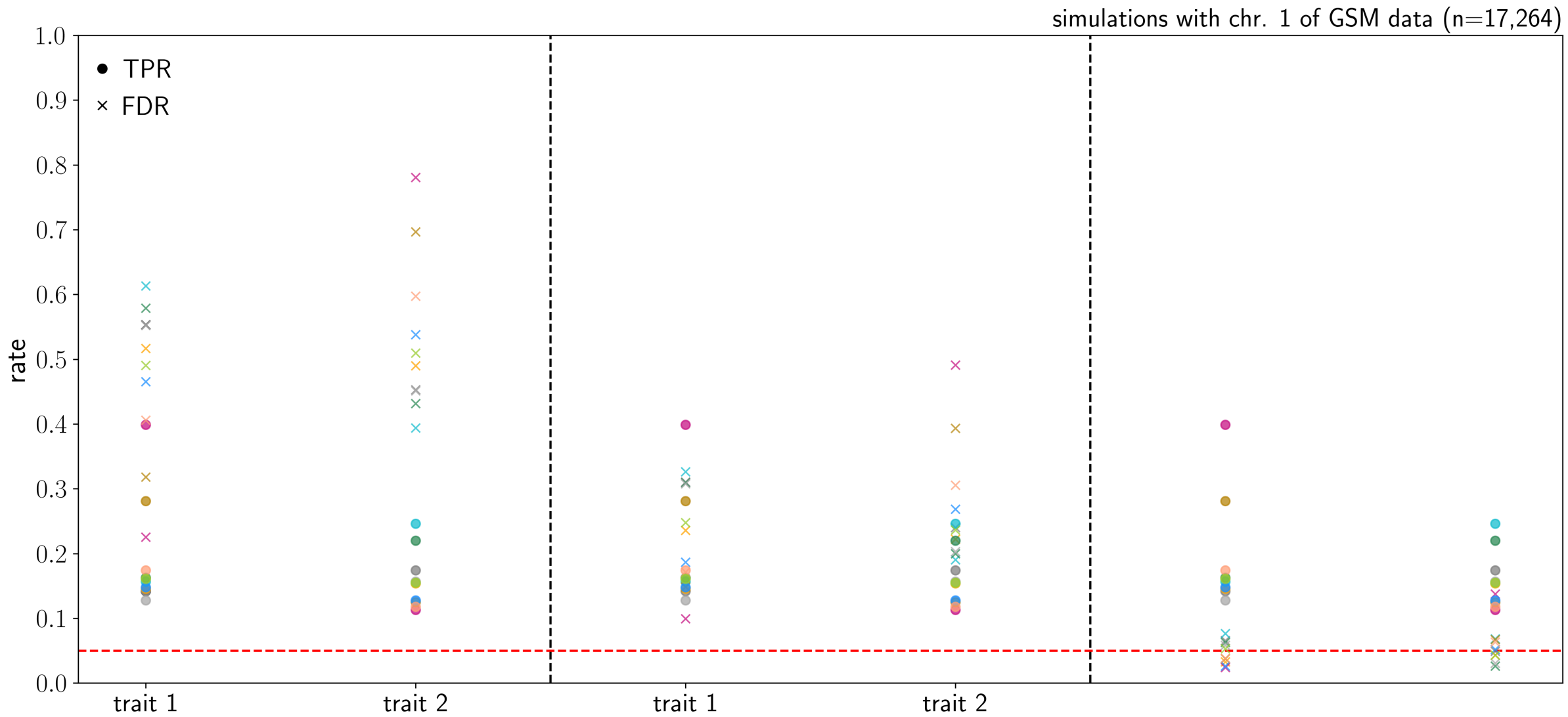

Figure S9: True positive rate (TPR) and false discovery rate (FDR) when simulating traits independently with a Gaussian with mean 0 and variances 0.5 for 1000 causal probes each. Probes are associated for a trait when posterior inclusion probability is bigger 0.95, and the mean posterior effects are 0 standard deviations (SD) (left), 1SD (middle) and 2SD (right) away from 0. The selection based on the strength of posterior effects is needed to control FDR. The red dashed line indicates an FDR threshold of 5%.

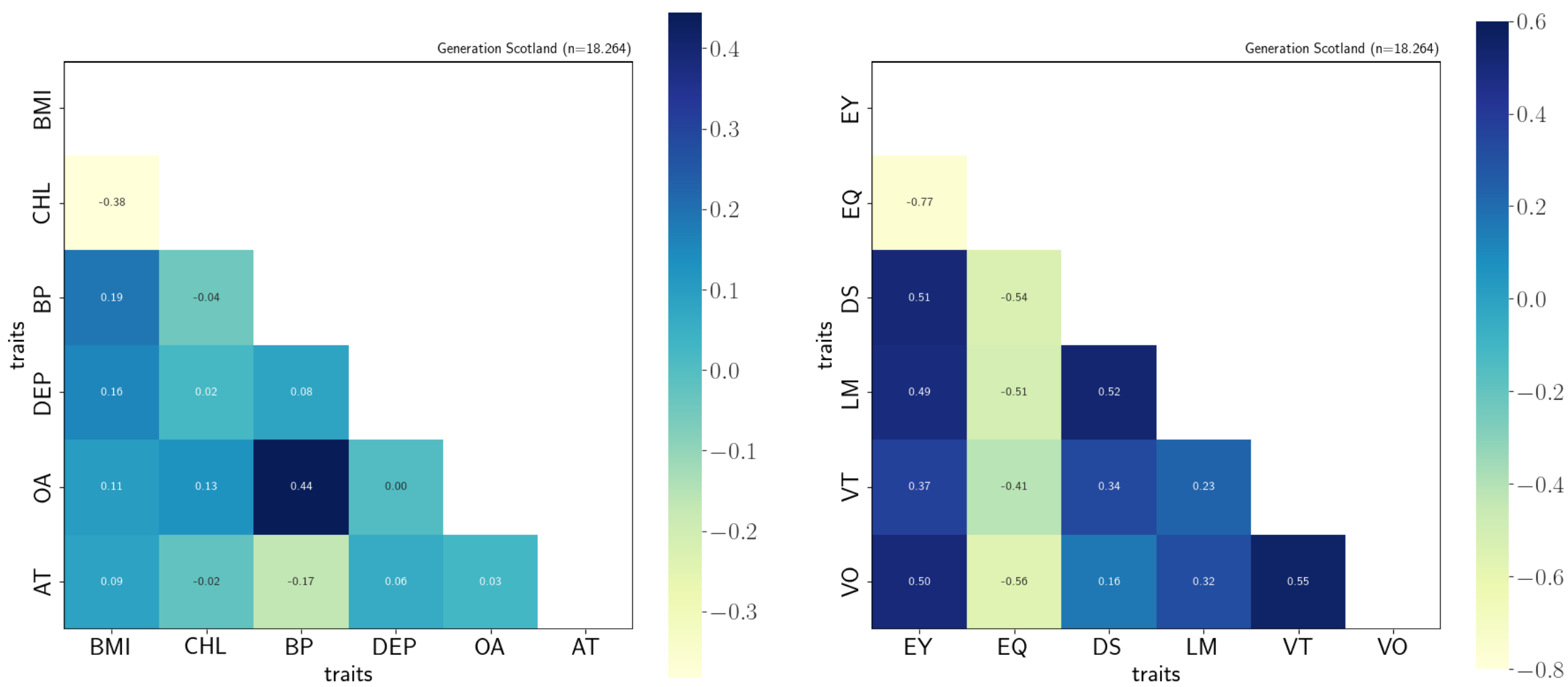

Figure S10: Correlations calculated from the posterior (co)variances for (left) body mass index (BMI), ratio of high density lipoprotein over total cholesterol (CHL), high blood pressure (BP), depression (DEP), osteoarthritis (OA) and asthma (AT) and (right) years in education (EY), highest qualification in education (EQ), digit symbol (DS), logical memory (LM), verbal fluency (VT) and vocabulary (VO) tests.

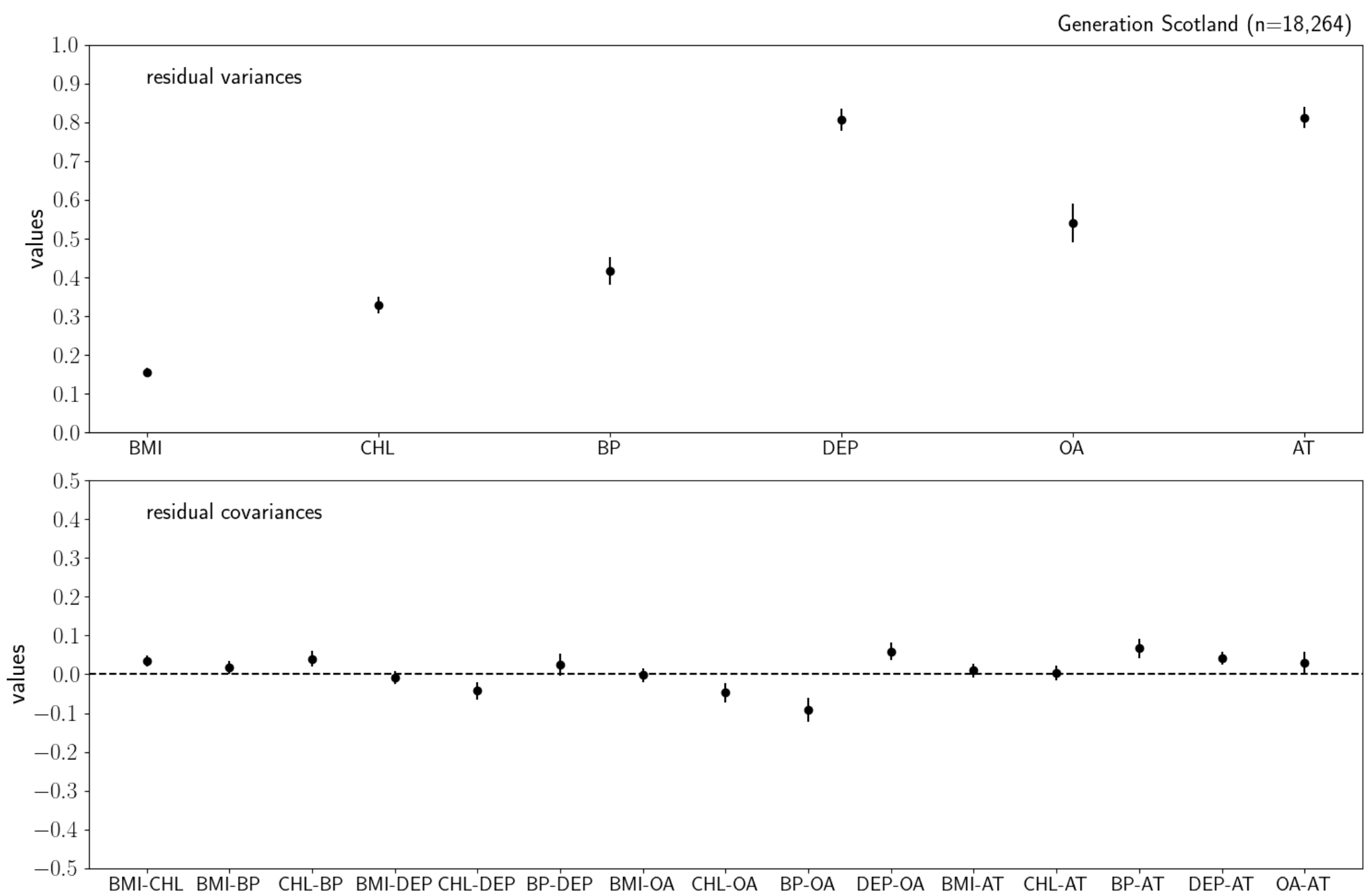

Figure S11: Estimated residual variances (top) and covariances (bottom) for body mass index (BMI), ratio of high density lipoprotein over total cholesterol (CHL), high blood pressure (BP), depression (DEP), osteoarthritis (OA) and asthma (AT) in the Generation Scotland methylation data using 18,624 individuals and 831,349 probes. The error bars represent the 95% credible interval.

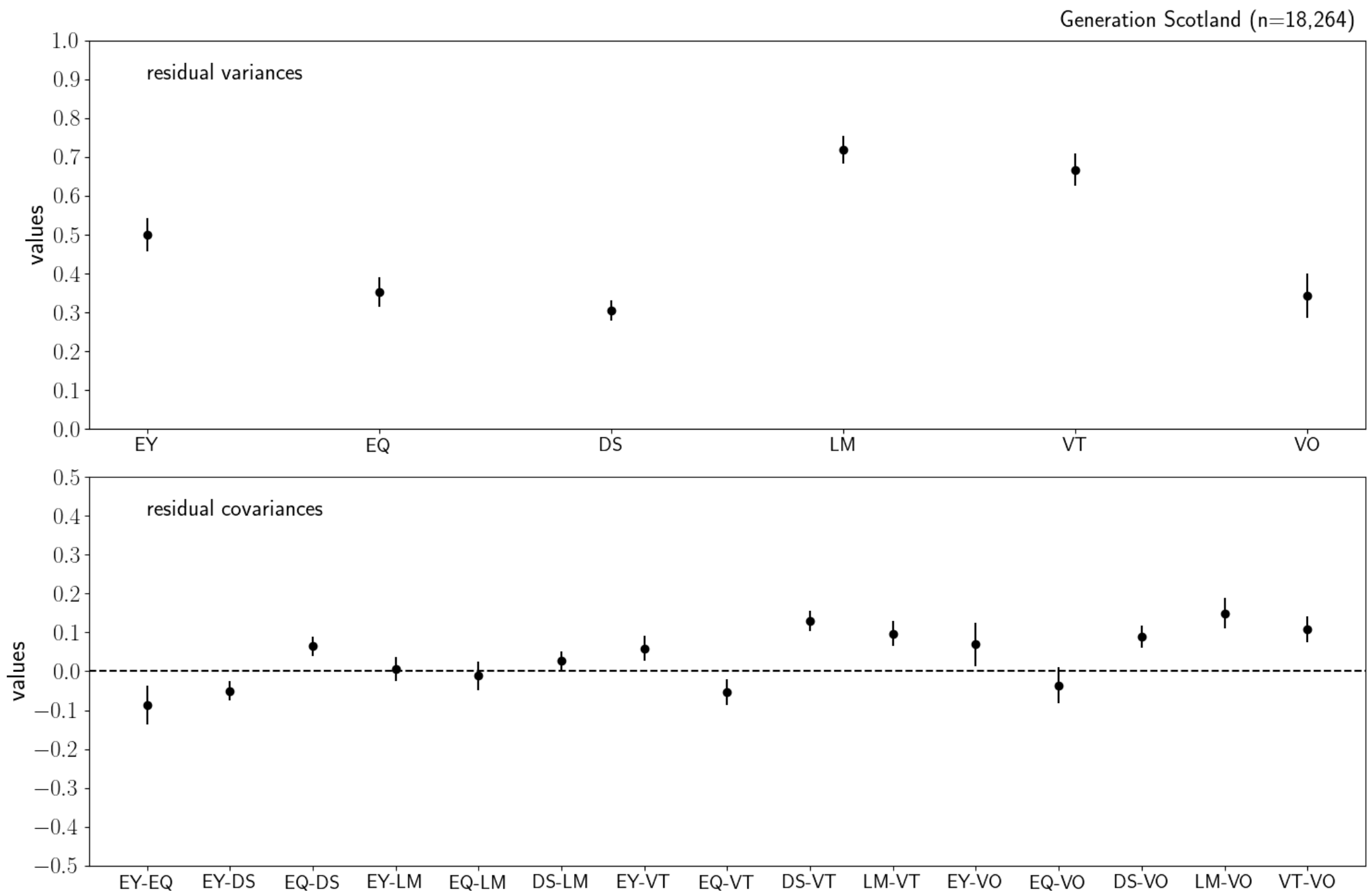

Figure S12: Estimated residual variances (top) and covariances (bottom) for years in education (EY), highest qualification in education (EQ), digit symbol (DS), logical memory (LM), verbal fluency (VT) and vocabulary (VO) tests in the Generation Scotland methylation data using 18,624 individuals and 831,349 probes. The error bars represent the 95% credible interval.

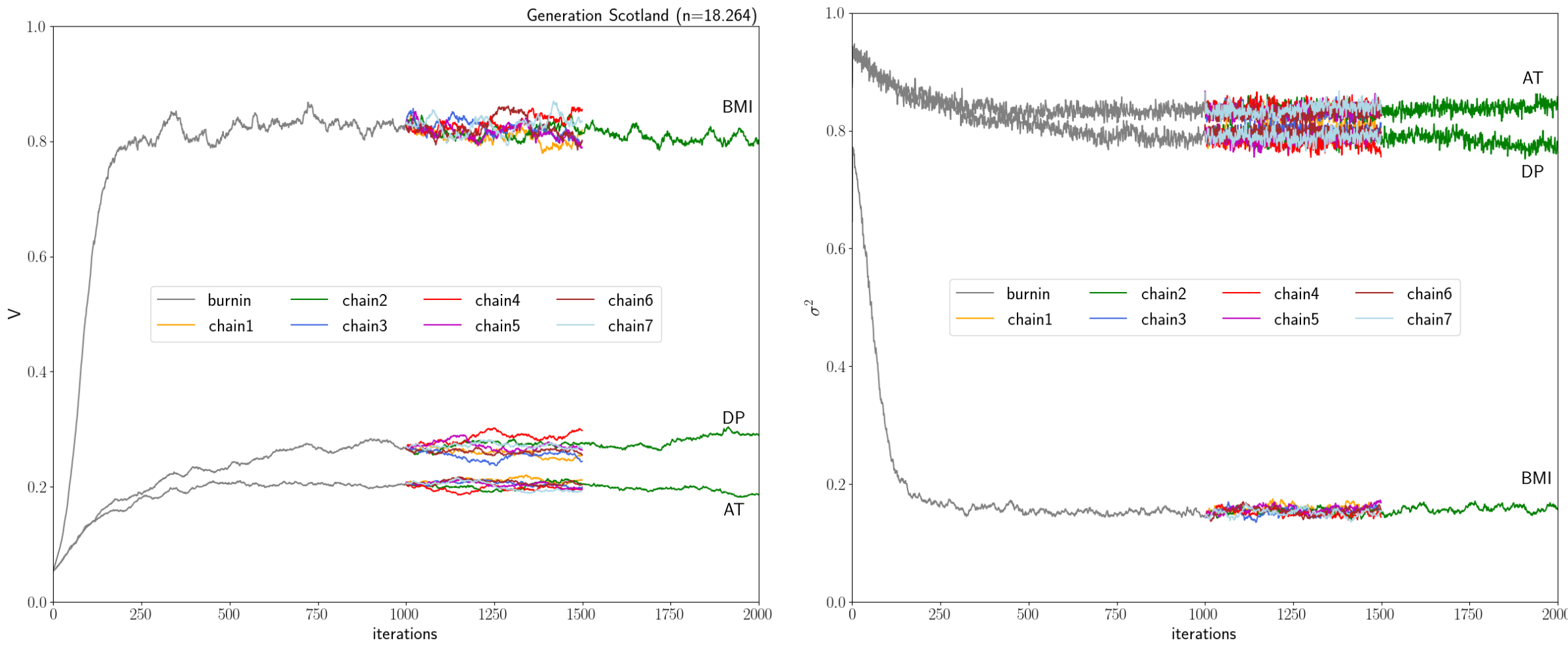

Figure S13: Variances (left) and residual variances (right) as function of iterations for body mass index (BMI), depression (DEP), and asthma (AT) in Generation Scotland methylation data using 18,624 individuals and 831,349 probes. After the burn-in period (1000 iterations), multiple chains are started.
